# Management of differentiated thyroid cancer through nuclear medicine facilities during Covid-19 emergency: the telemedicine challenge

**DOI:** 10.1007/s00259-020-05041-0

**Published:** 2020-09-23

**Authors:** Michele Klain, Carmela Nappi, Simone Maurea, Marina De Risi, Fabio Volpe, Elisa Caiazzo, Leandra Piscopo, Mariarosaria Manganelli, Martin Schlumberger, Alberto Cuocolo

**Affiliations:** Department of Advanced Biomedical Sciences, University Federico II, Naples, Italy

**Keywords:** Covid-19, SARS-CoV-2, Thyroid cancer, ^131^I, Southern Italy, Telemedicine

## Abstract

**Purpose:**

To investigate whether a telemedicine service (TMS) carried out during the Covid-19 pandemic impacted on management of patients with differentiated thyroid cancer (DTC).

**Methods:**

We retrospectively reviewed the number and the findings of outpatient visits in DTC subjects referred between March 11, 2020, and May 31, 2020, during the Covid-19 pandemic at the Radiometabolic Unit of the University of Naples Federico II. Office visits scheduled in March and May 2020 were converted in teleconsultation reaching all patients planned for an in-ward access to advise them to use the TMS for all clinical necessity. The number and the findings of DTC patients evaluated by in-ward access in the corresponding period of 2019 were also assessed for direct comparison.

**Results:**

The number of outpatient visits performed by TMS during the pandemic (*n* = 445) and by in-ward access in the corresponding period of 2019 (*n* = 525) was comparable with only 15% of outpatient evaluations missed.

**Conclusions:**

Our findings demonstrate the utility of telemedicine tools to avoid the potential negative impact of interruption or postponement of diagnostic and/or therapeutic procedures. Therefore, investments in medical network system development, including the implementation of telehealth approaches, should be encouraged at national and international levels.

## Introduction

From March 11, 2020, when the World Health Organization characterized the novel coronavirus Covid-19 outbreak as a pandemic, healthcare services were suddenly called to deal with a number of new critical issues [1]. On the one hand, the need to fight Covid-19 to reduce the mortality risk related to the virus infection [2] has rapidly emerged as a priority goal redirecting all available healthcare resources to this emergency response, and on the other hand, the access to medical facilities to manage acute and chronic diseases not related to Covid-19 has been subjected to an unplanned challenge. As regards to oncological units, the need to maintain the planned therapeutic management and surveillance has requested the identification of new monitoring strategies, using all available tools, including new technologies [3]. It should be also taken into account that oncological patients have a higher risk of mechanical ventilation, intensive care unit admission, or death compared with patients without cancer [4].

Although patients with differentiated thyroid cancer (DTC) have an excellent overall prognosis [5], distant metastases occur in 10% or even less of DTC patients and require an early and appropriate treatment to improve their prognosis and also need an active surveillance between treatment courses to detect progression and potential complications. Hence, in order to maintain the medical assistance of this patient’s category, a telemedicine service (TMS) by converting scheduled office visits to teleconsultations has been set up in medical units all over the world [6]. These visits can be conducted on distance, limiting both travel and exposure and permitting uninterrupted care of patients, and were proposed for the management of thyroid cancer in nuclear medicine departments [7].

Italy was among the first countries hit by the Covid-19 emergency, experiencing both virus clinical manifestations and virus containment measures imposed by the Italian Government to limit contagion spread [8]. While Southern Italy regions reported lower rates of Covid-19-related mortality than Northern Italy regions, lockdown measures affected clinical routine requiring rapid and effective alternative strategies to keep the medical engine going. The Radiometabolic Therapy Unit of the University Hospital Federico II promptly switched all scheduled nuclear medicine in-ward visits to teleconsultations as the first approach to medical assistance, leaving an on-site access only when strictly required.

The aim of the present study was to analyze whether a TMS carried out in our department during lockdown imposed by the Italian Government from March 11, 2020, to May 31, 2020, impacted the management of DTC patients, in comparison with data obtained during the same period of the year 2019 that were obtained only by in-ward access of outpatient examinations.

## Methods

### Study protocol

We evaluated all DTC patients who were referred to the Radiometabolic Therapy Unit of the University Hospital Federico II during the Covid-19 outbreak between March 11, 2020, and May 31, 2020, and those referred during the corresponding period of the year 2019. All patients included in the study were scheduled for an in-ward access for (a) a follow-up examination after initial treatment with total thyroidectomy and radioactive iodine (RAI) therapy or (b) a first examination after total thyroidectomy to consider the need for a RAI therapy according to the American Thyroid Association (ATA) guidelines [9]. Demographic and clinical information were prospectively collected in a database, consisting of age, sex, and post-operative histopathological data including tumor histology, lymph node involvement or distant metastasis, and RAI administration data. At the time of the most recent consultation, through both in-ward access and TMS, serum thyroglobulin (Tg) and thyroid-stimulating hormone (TSH) levels on L-thyroxine therapy, neck ultrasound findings, and eventual other imaging results were also collected. During follow-up, biochemical and ultrasound tests are usually performed outside the Radiometabolic Therapy Unit and results are brought at the follow-up appointment. In case of a discrepancy with the clinical records, before planning any further therapy change, further biochemical tests and imaging are performed at our Institution.

This study complies with the Declaration of Helsinki. The review committee of our Institution approved the study, and all patients gave their informed consent.

### Telemedicine service

All office visits scheduled between March 11, 2020, and May 31, 2020, were converted in teleconsultations by reaching all patients planned for an in-ward access to advise them to use the TMS. To avoid any possible social disparities regarding the ability to use different levels of technology, several tools have been used, including two active phone numbers, a fax number, and an e-mail address to receive biochemical and imaging data and to send out teleconsultation reports. Due to the short time to set up the service, the video conference method was not used. For follow-up examinations, all patients were reached by a phone call. In case of unremarkable findings, patients were scheduled for another follow-up teleconsultation 6 months later; in case of biochemical and/or ultrasound abnormalities, patients were asked to perform further imaging tests including chest and neck computed tomography with contrast, neck magnetic resonance, or ^18^F-FDG whole body positron emission tomography/computed tomography according to case-specific clinical suspicion. For patients requiring further treatment, an in-ward consultation was scheduled before hospitalization. As regards to the first post-operative examination, on the basis of postoperative risk stratification [9] patients for whom RAI treatment was not recommended were referred to their clinical physician for follow-up. Only in the case of an indication for RAI treatment, an in-ward consultation was scheduled before the hospitalization. All data regarding the patient’s evaluation were electronically stored on a cloud computing drive (Google) and updated in the patient’s individual clinical record.

### In-ward access

As regards to the 2019 months corresponding to the 2020 lockdown, all patients were visited by in-ward access. During the 2020 pandemic Covid-19 outbreak, if the attending physician, after a teleconsultation, deemed that a more detailed evaluation was necessary, patients were encouraged to schedule an in-ward consultation. In this case, patients were advised to follow all measures to prevent Covid-19 transmission (e.g., social distance, frequent handwashing, use of face masks in the hospital except in the hospitalization room, no accompanying person). During the Covid-19 pandemic, telephone triage for patients who needed in-ward access was performed in order to identify symptomatic patients (e.g., fever, tiredness, dry cough). In addition, body temperature was measured at the time of in-ward access. In case of symptoms and in the presence of suspicious Covid-19 disease or of suspicious contamination, patients performed a pharyngeal swab. Of note, medical staff members were asked to wear personal protective equipment and to follow all measures to prevent Covid-19 transmission. All surfaces in the patient care area and floors were cleaned first with soap and water and then with a fresh solution of chlorine-based products twice daily and after each patient visit.

### Statistical analysis

Continuous data are expressed as mean ± standard deviation and categorical data as percentage. Student’s *t* test and *χ*^2^ test were used to compare the differences in continuous and categorical variables, respectively. Two-tailed *P* values < 0.05 were considered significant. Statistical analysis was performed with R software version 3.6.3 (R Foundation for Statistical Computing, Vienna, Austria).

## Results

In our Institution, 2 referring physicians, 3 residents, and one nurse are usually involved in in-ward consultations. During the pandemic, the medical staff were not reduced in numbers. Indeed, the teleconsultation, as regards to TMS set up from both sides including the patients and the referring physicians, data evaluation, communication of teleconsultation results, and data storage, required a significant workload that can be estimated to be 30–40 min as average time while the average time for in-ward consultation was 15–25 min.

A total of 445 TMS visits were performed during the pandemic, and as compared with the corresponding period of 2019 by in-ward access (*n* = 525), only 15% of outpatient evaluations were missed. A 28% decrease in the number of first accesses for newly diagnosed DTC cases was observed, from 75 in 2019 to 54 during the corresponding period in 2020.

While the mean age and the percentage of male gender during the pandemic did not differ compared with the corresponding period of 2019 (50 ± 15 vs. 49 ± 14 years and 23% vs. 20% male gender respectively, both *P* = NS), the proportion of patients with classical papillary histology was significantly lower during pandemic compared with those evaluated in the corresponding months of 2019 (60% vs. 68%, *P* < 0.02).

Baseline characteristics of the overall population according to first evaluation and follow-up examinations during the Covid-19 pandemic and during the corresponding months of 2019 are shown in Tables 1 and 2. The proportion of patients with lymph node involvement evaluated at follow-up in 2019 was significantly higher than in those evaluated in 2020, but there was no statistical difference in the other analyzed characteristics.

**Table 1:** Patient characteristics at first visit during the Covid-19 pandemic and during the corresponding period of 2019

	2020 (*n* = 54)	2019 (*n* = 75)	*P* value
Age (year)	45 ± 16	46 ± 15	0.67
Male gender, *n* (%)	13 (24)	21 (28)	0.62
Classical papillary histology, *n* (%)	33 (59)	56 (74)	0.10
Lymph node involvement, *n* (%)	15 (27)	16 (21)	0.40
Distant metastasis, *n* (%)	0	0	NA
TSH (mUI/mL)	1.4 ± 1.3	1.9 ± 1.3	0.09
Tg (ng/mL)	26 ± 144	13 ± 74	0.54
Time to RAI treatment (days)	42 ± 3	45 ± 2	0.39

Values are expressed as mean value ± standard deviation or as number (percentage) of subjects

*TSH*, thyroid stimulating hormone; *Tg*, thyroglobulin; *RAI*, radioactive iodine

**Table 2:** Patient characteristics at follow-up visit during the Covid-19 pandemic and during the corresponding period of 2019

	2020 (*n* = 391)	2019 (*n* = 450)	*P* value
Age (year)	51 ± 14	50 ± 14	0.43
Male gender, *n* (%)	91 (23)	83 (18)	0.08
Classical papillary histology, *n* (%)	239 (61)	303 (67)	0.07
Lymph node involvement, *n* (%)	39 (10)	75 (17)	0.005
Distant metastasis, *n* (%)	3 (1)	5 (1)	0.73
TSH (mUI/mL)	2.05 ± 10.2	3.6 ± 23.3	0.21
Tg (ng/mL)	6.5 ± 80.1	1.5 ± 18.2	0.20

Values are expressed as mean value ± standard deviation or as number (percentage) of subjects

*TSH*, thyroid stimulating hormone; *Tg*, thyroglobulin

Although among newly DTC diagnosed cases, there was a slightly higher proportion of patients requiring RAI therapy after surgical treatment during the Covid-19 pandemic compared with patients evaluated during the corresponding period of 2019, a statistical significance was not reached (85% vs. 71%, *P* = NS).

Results of follow-up visits obtained in outpatients by TMS during the Covid-19 pandemic and by in-ward access during the corresponding period of 2019 were analyzed according to three subgroups of patients: (1) those requiring a further RAI therapy or surgical treatment (8/450 vs. 7/391), (2) those requiring further diagnostic tests (28/450 vs. 34/391), and (3) those with unremarkable findings (414/450 vs. 350/391). The proportion of patients in each subgroup did not differ between the two considered periods (all *P* = NS).

Of note, all patients requiring RAI therapy, including those 7 of the 391 patients evaluated during follow-up and those 46 of the 54 patients evaluated post-operatively, were then asked to schedule an in-ward consultation. In addition, all scheduled RAI therapies during the Covid-19 emergency were performed after telephone triage and pharyngeal swab. No patient treated during the pandemic demonstrated Covid-19. In particular, 46 DTC patients were treated with RAI during the 2020 emergency and this number was not different from the 54 patients treated with RAI in 2019.

With regard to lung disease, which is the most frequent link to Covid-19 infection, the number of patients with lung metastasis evaluated by chest imaging during the Covid-19 pandemic did not differ compared with the corresponding period of 2019 (5/525 vs. 3/445, *P* = NS). Of note, all patients with lung metastasis were already included on the disease active surveillance program and no suspicious interstitial pneumonia infection was observed.

## Discussion

Our experience demonstrates that, despite the virus containment measures during the Covid-19 pandemic, the number of DTC patients’ evaluations performed by TMS was only 15% less as compared with the in-ward examinations in the corresponding months of 2019. Interestingly, this decreased percentage was 28% for first access examinations after thyroidectomy. This finding is not surprising given the general postponement of all surgical interventions.

As regarded to chest imaging performed in patients with lung disease, no suspicious interstitial pneumonia infection was observed. Our finding is consistent with Maurea et al. results [10] who reported no positive cases for Covid-19 infection in our same Department confirming that the Covid-19 outbreak has been so far contained in these patients from Southern Italy.

The current investigation first shows the utility of telemedicine tools at the time of Covid-19 in a nuclear medicine department for RAI therapy management. The use of TMS allowed both physicians and patients to communicate regardless of restrictive measures keeping up the therapeutic alliance and assuring active surveillance on disease course, suggesting that such a tool may successfully be implemented in routine clinical practice.

According to clinical practice guidance proposed by Vrachimis et al. [7], in order to avoid the detrimental impact of interruption or postponement of diagnostic and/or therapeutic procedures, we set up a TMS by converting all in-ward access visits scheduled within March 11, 2020, and May 31, 2020, in teleconsultation appointments. Telemedicine, defined as the provision of medical care remotely by audio-visual technology is not a novel concept [11]. Its applications vary from remote evaluation of imaging data acquired on-site to overcome social and geographic boundaries [12] with the benefit from expert opinion, to interdisciplinary case board examinations with off-site physicians [13] and teleconsultation between patients and referring physicians.

In particular, nuclear medicine departments, consisting in diagnostic and therapeutic units, were challenged to experience different approaches according to different medical modalities [14–17]. The necessity to optimize resources, keeping the radionuclide therapy service performances unchanged, required an upstream organization including the possibility to earmark outpatients’ visits to teleconsultation. Yet, it should be kept in mind that TMS required a higher workload than in-ward consultation. However, TMS performance has been affected by the lack of standardization and the short time available to set up the service that could be significantly improved from this initial experience.

The need to promptly find out alternative solutions speeded up TMS use highlighting not only the advantages of such a tool but also the pitfalls and criticism to be overcome in the future. Telecommunication strategy implementation should take into account the need to standardize the practice of TMS by providing medical staff training to ensure appropriate disease management and patient satisfaction feelings. In addition, explicit patient consent to share medical history data and patient privacy protection should be assured by using dedicated telehealth platforms with documented high confidentiality levels. It should be also considered that, if on one hand remote consultation may pave the way to ambitious perspectives, on the other it may imply new challenges regarding existing lacks not only on network communication systems but also in consideration of psychological implications. Of note, thyroid cancer patients like any other oncological patient may suffer by impaired affective and cognitive functioning needing to physically interface to the doctor to feel reassured [18].

Although the video call method has been not used during the Covid-19 emergency due to short time available for setting everything up, the implementation of a dedicated software providing video call tool could be worthwhile assuring a face-to-face approach even on distance.

It should be taken into account that teleconsultation has been used only for first-line approach. Indeed, in case of treatment change or to communicate recurrence of disease, patients were asked to schedule an in-ward consultation before any further treatment. Thus, TMS can be a useful first-line tool but a face-to-face approach should be highly recommended in case of disease recurrence or progression to provide the most comfortable environment leaving a safe space to emotional disease components. On the basis of the reported experience, TMS should be intended especially for active surveillance of DTC patients after RAI treatment, and an in-ward access being provided only for those showing abnormalities. An in-ward access should also be directly scheduled for the post-operative consultation when a RAI treatment is indicated to build up a strong therapeutic alliance.

Finally, from an economical viewpoint, once implemented and standardized, TMS would provide several benefits. From the patient side, the possibility to obtain medical consultation on distance would avoid transports and reduce the need to take a day-off from work to reach the hospital. It could be also possible to modulate the time to send out medical data according to patient preferences obtaining teleconsultation results directly at home 24/7. From the health service side, despite initial costs to build up a fully efficient TMS, such a tool would probably provide reduced costs on long term, but this needs a medico-economic evaluation.

## Conclusion

In the light of the Covid-19 experience, investments on medical network system development, including telehealth approaches implementation, should be encouraged at the national and international level, recognizing reimbursement, building up dedicated virtual report system to safely store and share medical information, prescriptions, and further test recommendations without neglecting the role of the in-person meeting as a valuable component for a successful therapeutic approach.
